# GLP-1 receptor agonists at the crossroads of diabetes, hepatic steatosis, and hepatocellular carcinoma

**DOI:** 10.1007/s11739-026-04456-1

**Published:** 2026-07-28

**Authors:** Alfredo Vozza, Filippo Biagi, Chiara Raggi, Francesco Vizzutti, Fabio Marra

**Affiliations:** 1 Dipartimento di Medicina Sperimentale e Clinica, University of Florence, Largo Brambilla, 3, 50134 Florence, Italy; 2 Interdisciplinary Department of Medicine, University of Bari “Aldo Moro”, Bari, Italy; 3 Internal Medicine Unit, SS. Annunziata Hospital, Taranto, Italy

**Keywords:** GLP-1RA, MASLD, Type 2 diabetes, HCC, MASH, Hepatic decompensation

## Abstract

The epidemiology of hepatocellular carcinoma (HCC) is increasingly shaped by the expanding burden of type 2 diabetes (T2D), obesity, and metabolic dysfunction-associated steatotic liver disease (MASLD). Glucagon-like peptide-1 receptor agonists (GLP-1RAs), initially developed as glucose-lowering agents, have emerged as systemic metabolic therapies with effects extending beyond glycemic control, including weight reduction, cardiovascular and renal protection, and improvement of steatotic liver disease. This review summarizes current evidence on the potential role of GLP-1RAs in modifying the natural history of MASLD and reducing HCC risk. Clinical trials consistently support beneficial effects on steatosis and metabolic dysfunction-associated steatohepatitis, particularly in non-cirrhotic patients, whereas effects in established cirrhosis appear less consistent. Real-world studies and target trial emulations suggest that GLP-1RA use may be associated with lower rates of hepatic decompensation, cirrhosis progression, and incident HCC, although the magnitude and robustness of these associations vary according to disease stage, comparator therapy, exposure definition, and outcome hierarchy. The strongest apparent benefit is generally observed against insulin or sulfonylureas, while differences are attenuated in comparisons with other metabolically favorable agents, such as SGLT2 inhibitors. Mechanistic data support indirect hepatoprotective actions mediated by reduced insulin resistance, lipotoxicity, inflammation, fibrogenesis, adipose–liver crosstalk, and tumor-promoting pathways. Overall, GLP-1RAs represent promising disease-modifying agents in metabolic liver disease, but dedicated prospective studies are required to establish causality and define their role in HCC prevention.

## Introduction

Hepatocellular carcinoma (HCC) is the most common primary liver malignancy and represents the fourth leading cause of cancer-related mortality worldwide [1]. While chronic viral hepatitis has historically been the dominant etiological factor, the epidemiological landscape of HCC is shifting. The global decline in the incidence of viral hepatitis, due to widespread vaccination and effective antiviral therapies, has unmasked the growing contribution of metabolic risk factors, including obesity, type 2 diabetes (T2D), and metabolic dysfunction-associated steatotic liver disease (MASLD). In contemporary Western cohorts, MASLD has emerged as one of the leading causes of HCC and the prevalence of metabolic-related HCC is expected to rise further in the coming decades [2–4].

MASLD, which affects nearly one quarter of the global adult population, is tightly linked to T2D, where the prevalence of MASLD exceeds 60%, and a significant proportion of patients progresses to advanced fibrosis and cirrhosis [5, 6]. T2D is an established independent risk factor for both liver-related and all-cause mortality. Epidemiological studies demonstrate that individuals with T2D have a twofold to threefold increased risk of developing HCC compared with non-diabetic subjects, irrespective of traditional risk factors such as alcohol consumption or viral hepatitis [7]. This excess risk is multifactorial: chronic hyperglycemia, insulin resistance, and hyperinsulinemia drive hepatic steatosis and steatohepatitis, promote fibrogenesis, and sustain a pro-inflammatory and pro-oncogenic hepatic microenvironment. Furthermore, T2D is frequently accompanied by obesity, dyslipidemia, and systemic inflammation, amplifying the risk of progressive liver disease and malignant transformation [8]. A peculiar characteristic of HCC in the context of MASLD is the high frequency of cancers arising in non-cirrhotic livers, challenging the traditional paradigms of hepatocarcinogenesis and complicating surveillance strategies [9, 10]. Moreover, T2D and MASLD synergistically accelerate the risk of adverse liver outcomes, including decompensation, need for transplantation, and HCC [11, 12].

The past 2 years have witnessed the appearance of new therapeutic strategies that are effective for the treatment of MASH. Resmetirom, a thyroid *β*-receptor agonist, has been the first drug to be approved for the treatment of MASH with fibrosis [13]. Additionally, therapeutic strategies that predominantly target the metabolic drivers of MASLD are being developed. Glucagon-like peptide-1 receptor agonists (GLP-1RAs) have revolutionized the management of T2D, improving glycemic control, promoting weight loss, and conferring robust cardiovascular and renal protection [14, 15]. Recent lines of evidence indicate that their pleiotropic metabolic and anti-inflammatory effects extend to the liver, potentially altering the natural history of MASLD and possibly modulating the risk of HCC [16–19]. Given the paucity of effective chemopreventive strategies in this field, the exploration of GLP-1RAs as disease-modifying agents in the context of liver carcinogenesis is both timely and of high clinical relevance.

In this review, we summarize the current evidence on the role of GLP-1RAs in patients with T2D and MASLD, with particular focus on their potential to mitigate the risk of HCC. To this end, a comprehensive literature search was conducted in PubMed/MEDLINE to identify relevant studies evaluating the role of GLP-1RAs in patients with T2D and MASLD, with particular emphasis on HCC and liver-related outcomes. The search covered articles published up to May 2026 and was performed using multiple combinations of the following keywords: type 2 diabetes, T2D, diabetes mellitus, MASH, MASLD, NAFLD, NASH, steatotic liver disease, hepatocellular carcinoma, HCC, GLP-1 receptor agonists, GLP-1RA, glucagon-like peptide-1 receptor agonists, liraglutide, exenatide, semaglutide, dulaglutide, and tirzepatide, hepatic decompensation and liver outcomes. We included randomized controlled trials, observational cohort studies, target trial emulations, meta-analyses, and relevant translational and mechanistic studies. Articles focusing on non-metabolic etiologies of liver disease or lacking liver-specific outcomes were excluded. Additional references were identified through manual screening of bibliographies of key articles. Only studies published in English were considered.

Terminology for metabolic liver disease has evolved over time (NAFLD/NASH, MAFLD, and MASLD/MASH) [20]. Although these definitions are not fully identical, only the terms MASLD and MASH are used throughout this review for consistency. The review is structured as follows: we first examine the metabolic and hepatic effects of GLP-1RAs, and then critically appraise clinical studies evaluating their association with HCC incidence and prognosis across different populations and comparator therapies. Finally, we discuss the mechanistic pathways underlying their potential hepatocarcinogenic protection, together with key unmet needs and future research directions.

## GLP-1 receptor agonists: effects on type 2 diabetes and MASLD

GLP-1RAs have emerged as a cornerstone in the management of T2D, owing to their ability to improve glycemic control while simultaneously promoting weight loss. These agents mimic the effects of the incretin hormone GLP-1, enhancing glucose-dependent insulin secretion, suppressing glucagon release, delaying gastric emptying, and increasing satiety [21, 22]. Beyond glucose control, GLP-1RAs exert profound systemic effects. Large cardiovascular outcome trials have demonstrated reductions in major cardiovascular events, cardiovascular mortality, and progression of chronic kidney disease in high-risk individuals [14, 15]. These pleiotropic effects highlight the potential of GLP-1RAs as disease-modifying agents that act across multiple organ systems affected by metabolic dysregulation.

### GLP-1RAs and MASLD: clinical trial evidence

MASLD, closely intertwined with T2D, is characterized by hepatic steatosis and insulin resistance. In the more progressive forms, indicated as MASH, lipotoxicity induces hepatocellular damage, chronic inflammation, and fibrosis, eventually leading, in predisposed individuals, to cirrhosis and its complications [23–25]. Based on the potential ability of GLP-1RAs to affect several of the pathophysiological drivers of MASLD/MASH, their effects have been studied in different clinical trials. In the pivotal LEAN trial, liraglutide significantly increased the histological resolution of nonalcoholic steatohepatitis (MASH) compared to placebo, although the effects on fibrosis were modest [26]. More recently, semaglutide has been intensively investigated for the treatment of non-cirrhotic patients with MASH. In a phase II trial, it significantly increased the rate of MASH resolution without improving fibrosis stage [27]. In a subsequent phase III program in individuals with F2–F3 fibrosis, once weekly, 2.4 mg semaglutide achieved significant histologic benefits [19]. In contrast, among patients with compensated cirrhosis, semaglutide did not improve fibrosis or induced MASH resolution compared with placebo [28]. Complementary evidence from imaging and biomarker analyses reinforces these results, as GLP-1RA treatment reduced liver fat content as assessed by MRI-PDFF, lowered serum aminotransferases, and improved non-invasive markers of liver disease activity and fibrogenesis, including the enhanced liver fibrosis (ELF) and FibroScan-AST (FAST) scores and PRO-C3 levels [19, 26, 27, 29]. Taken together, these findings indicate that GLP-1RAs have a significant impact on MASH and fibrosis in earlier stages of disease, thereby potentially interrupting the fibrogenic cascade leading to cirrhosis.

### Real-world evidence for the impact on liver outcomes in studies designed for patients with T2D

**Fig. 1:**
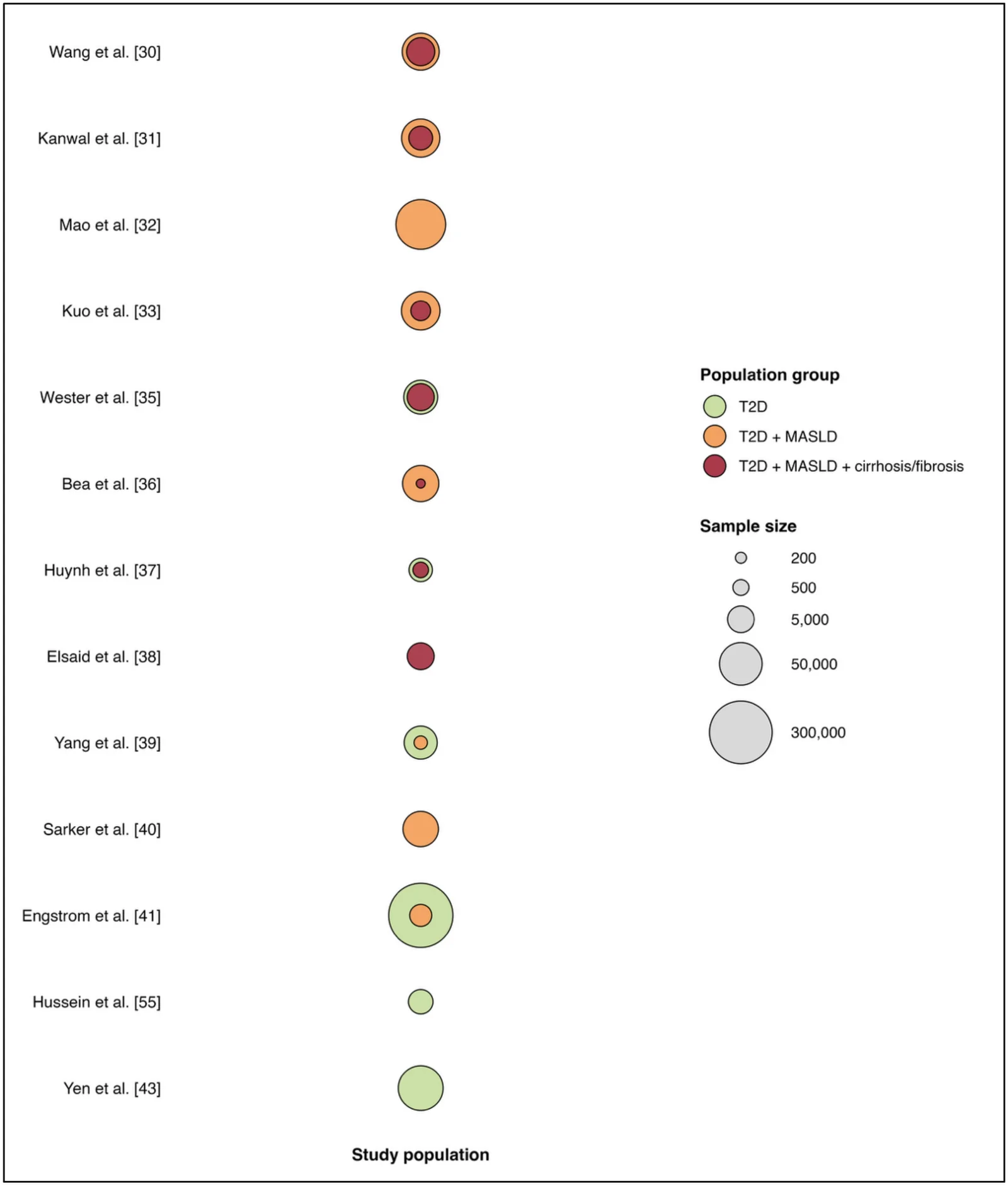
Population heterogeneity across observational studies. Concentric bubble plot showing the heterogeneity of study populations across observational studies. Each study is represented by concentric circles, with color identifying the population group considered within the study, including T2D, T2D with MASLD, and T2D with MASLD plus cirrhosis or advanced fibrosis. Circle size is proportional to study population size and is displayed using a square-root scale applied globally across studies. This representation highlights the marked variability in the underlying populations contributing to the observational evidence base, from broad diabetic cohorts to progressively more selected liver-enriched populations, thereby emphasizing the limited comparability of available findings

The current lack of long-term information on the possible effects of GLP-1-RAs on the appearance of liver outcomes such as progression to cirrhosis and its complications, in specifically designed trials, makes it useful to review evidence deriving from trials designed for patients with T2D (Table 1 and Fig. 1). Nonetheless, these lines of information are relevant to MASLD, considering the high prevalence of this condition in the diabetic population.

**Table 1:** Main characteristics of studies included in the review

Study	Study type	Population	Sample size	Treatment received	Length of study	Primary outcome	Additional outcome
Wang et al 2024 [29]	Observational retrospecrive (subgroup analysis)	T2D + MASLD/MASH	24975	- Cases: GLP-1 RAs - Controls: insulins, metformin, DPP4-i, SGLT2-i, SUs, TZDs	5 years	HCC: - *vs insulin = 0.34 (0.17–0.70)* - vs metformin = 1.21 (0.75–1.95) - vs DPP4-Is = 1.11 (0.77–1.60) - vs SGLT2-Is = 0.98 (0.64–1.51) - vs SUs = 1.03 (0.72–1.49) - vs TZDs = 0.99 (0.60–1.62)	Hepatic decompensation events^a^: - *vs insulin = 0.31 (0.25–0.39)* - vs metformin = 0.86 (0.74–1.01) - *vs DPP4-I = 0.76 (0.68–0.85)* - vs SGLT2-I = 0.87 (0.75–1.02) - *vs SU = 0.76 (0.67–0.85)* - vs TZD = 0.97 (0.79–1.15)
		T2D + liver fibrosis and cirrhosis	6588	- Cases: GLP-1 RAs - Controls: insulins, metformin, DPP4-i, SGLT2-i, SUs, TZDs		HCC: - *vs insulin = 0.27 (0.12–0.63)* - vs metformin = 1.14 (0.79–1.64) - vs DPP4-Is = 1.12 (0.84–1.51) - vs SGLT2-Is = 1.03 (0.72–1.49) - vs SUs = 0.86 (0.64–1.15) - vs TZDs = 1.48 (0.97–2.26)	Hepatic decompensation events^a^: - *vs insulin = 0.43 (0.34–0.55)* - vs metformin = 0.87 (0.76–0.99) - *vs DPP4-I = 0.81 (0.73–0.90)* - vs SGLT2-I = 0.96 (0.83–1.11) - *vs SU = 0.79 (0.71–0.88)* - vs TZD = 1.03 (0.88–1.21)
Kanwal et al 2024 [30]	Observational retrospecrive	T2D + MASLD	29,212	- Cases: GLP1-RAs - Controls: DPP4-i	2.9 years	*Cirrhosis = 0.86 (0.75–0.98)*	- Cirrhosis complication^b^ = 0.78 (0.59–1.04) - Hepatic decompensation = 0.75 (0.55–1.01) - HCC = 0.89 (0.40–2.01) - *All-cause mortality = 0.89 (0.81.0.98)*
		T2D + MASLD + cirrhosis	2904	- Cases: GLP1-RAs - Controls: DPP4-i	3.2 years	-Cirrhosis complications^b^ = 1.18 (0.77–1.81)	- Hepatic decompensation = 1.14 (0.72–1.82) - HCC = 1.41 (0.69–2.90) - All-cause mortality = 0.88 (0.73–1.06)
Mao et al 2025 [31]	Observational retrospecrive	T2D + MASLD	103,627	- Cases: GLP1-RAs - Controls: DPP4-i	2 years	- *HCC = 0.53 (0.39–0.71)* - *Cirrhosis = 0.91 (0.86–0.96)*	- Non-liver adverse event^c^
Kuo et al 2025 [32]	Observational retrospecrive	T2D + MASLD (4% cirrhosis)	30,352 (1214)	- Cases: GLP1-RAs - Controls: SGLT2-i	2.6 years	- *MALO*^*d*^* = 0.84 (0.73–0.97)*	- *Hepatic decompensation = 0.83 (0.71–0.96)* - HCC = 0.94 (0.63–1.42) - Liver transplantation = 1.21 (0.63–2.34) - All-cause mortality = 0.84 (0.75–0.94)
Wester et al 2024 [34]	Observational retrospecrive (subgroup analysis)	T2D + CLD (33.4% MASLD)	16,294 (5449)	- Cases: GLP1-Ras - Controls: no GLP1-RAs	5.3–6.3 years	- MALO^f^ = 1.41 (0.53–2.30)	-
Bea et al 2024 [35]	Observational retrospective	T2D + MASLD (0.8% cirrhosis)	22,550 (180)	- Case: SGLT2-Is - Controls: GLP1-RAs	2.1 years	- Hepatic decompensation^g^ = 0.93 (0.76–1.14)	- Ascites = 1.12 (0.88–1.43) - Oesophageal varices with bleeding = 0.80 (0.35–1.83) - *Liver transplant = 0.25 (0.09–0.70)* - *Hepatic failure = 0.71 (0.50–1.00)* - Liver- cause mortality = 0.45 (0.15–1.32) - All-cause mortality = 1.24 (0.90–1.72) - HCC = 0.75 (0.43–1.32)
Huynh et al 2023 [36]	Observational retrospective (subgroup analysis)	T2D + cirrhosis (17.4% MASH cirrosi)	2632 (458)	- Cases: GLP1-RAs + metformin - Controls: metformin	5 years	*- 5 year mortality risk = 0.13 (0.03–0.58)*	- ^h^Hepatic decompensation = 0.36 (0.11–1.15) - HCC = 0.62 (0.18–2.22)
Elsaid et al 2024 [37]	Observational retrospective	T2D + MASLD + cirrhosis	5296	- Cases: GLP1-Ras - Controls: no GLP1-RAs	1.3 years	- *MALO*^*i*^* = 0.64 (0.54–0.76)*	- *Hepatic decompensation*^*l*^* = 0.74 (0.61–0.88)* - *Portal hypertension = 0.73 (0.60–0.88)* - *HCC = 0.37 (0.20–0.63)* - *Liver transplantation = 0.24 (0.12–0.43)*
Yang et al 2024 [38]	Observational retrospective	T2D (2% MASLD)	14,342 (287)	- Cases: GLP1-Ras - Controls: Long acting insulins	3.5–4 years	- *Cirrhosis or HCC = 0.52 (0.35–0.79)*	- *Cirrhosis = 0.52 (0.33–0.81)* - HCC = 0.49 (0.18–1.29)
Sarker et al 2026 [39]	Observational retrospective	T2D + MASLD	20,228	-GLP1-Ras Controls: SGLT2i or DDP4i	2 years	HCC or Cirrhosis (1-year follow-up): -vs DPP4i = 0.79 (0.55–1.15) -*vs SGLT2i = 0.61 (0.42–0.88)* HCC or Cirrhosis (two years follow-up): -vs DPP4i = 0.80 (0.58–1.09) -vs SGLT2i = 0.76 (0.55–1.04)	HCC: -vs DPP41 = 0.20 (0.02–1.71) -vs SGLT2i = no events Cirrhosis: -vs DPP4i = 0.81 (0.59–1.10) -vs SGLT2i = 0.75 (0.55–1.03)
Engstrom et al 2023 [40]	Observational retrospective	T2D (1.2% MASLD/MASH)	335,483 (2013)	- Cases: GLP1-RAs - Controls: DPP4-i	3 years	- *Compensated and decompensated cirrhosis*^*m*^*, or HCC = 0.85 (0.75–0.97)*	- *Compensated and decompensated cirrhosis*^*m*^* = 0.85 (0.75–0.97)* - HCC = 1.05 (0.80–1.39)
Hussein et al 2025 [54]	Observational retrospective (subgroup analysis)	T2D	3364	-Cases: bariatric surgery - Controls: GLP1-RA	3.7 years	- NASH = 1.09 (0.89–1.36)	- Cirrhosis = 2.67 (1.39–5.10) - HCC = 0.98 (0.22–4.40) - All-cause mortality = 1.06 (0.68–1.08)
Yen et al 2024 [42]	Observational retrospective	T2D (no cirrhosis)	62,312	- Cases: GLP1-Ras - Controls: no GLP1-RAs	2.2 years	- *All‐cause mortality = 0.48 (0.43–0.53)* - *MACE = 0.92 (0.86–0.99)* - *Cardiovascular death = 0.57 (0.45–0.72)* - *Liver‐related death = 0.32 (0.13–0.75)* - Cirrhosis = 1.10 (0.88–1.37) - Hepatic failure = 0.92 (0.66–1.30) - HCC = 0.91 (0.59–1.40)	-
Pasta et al 2025 [16]	Meta-analysis	T2D	641,377	- Cases: GLP1-Ras - Controls: no GLP1-RAs	1.3–6 years	- *HCC = 0.42 (0.19–0.93)*	- *HCC (subset of cirrhosis patients) = 0.44 (0.25–0.76)* - Hepatic decompensation = 0.49 (0.19–1.26)
Passos et al 2025 [33]	Meta-analysis (subgroup analysis)	T2D (% MASLD not reported)	523,921 (-)	- Cases: GLP1-Ras - Controls: no GLP1-RAs	2.9 years	- HCC = 0.65 (0.28–1.48) - *Cirrhosis decompensation*^*e*^* = 0.79 (0.66–0.94)*	-
Shabil et al 2024 [41]	Meta-analysis	T2D	5,387,826	Cases: GLP1-Ras - Controls: no GLP1-Ras	1–15 years	- *HCC = 0.41 (0.28–0.55)*	- *vs no GLP1-RAS other than insulin = 0.47 (0.29–0.65)* - *vs insulin = 0.36 (0.16–0.56)*
Celsa et al 2025 [44]	Meta-analysis	T2D	1,467,220	- Cases: GLP1-Ras - Controls: no GLP1-RAs	3.5–6 years	- *MALO*^*n*^* = 0.71 (0.57–0.88)*	- *Hepatic decompensation = 0.70 (0.52–0.94)* - HCC = 0.82 (0.61–1.11)
Li et al 2026 [45]	Meta-analysis	T2D + MASLD	737,408	-GLP1-RA -Controls: non-GLP-1R, SGLT2i, SU, DPP4i	3 years	Liver related events^o^: -*vs non GLP1-RA = 0.79 (0.70–0.90)* -vs SGLT2i = 0.94 (0.85–1.04) -*vs DDP4i = 0.78 (0.68–0.89)* -*vs SU = 0.77 (0.69–0.86)*	*Liver related events (non asian region):* *-vs SGLT2i = 0.91(0.83–0.99)* Cirrhosis: -vs SGLT2i = 1.09 (0.91–1.3) HCC: -vs non-GP1-RAs = 0.72 (0.38–1.38) -SGLT2i vs GLP1-Ras = 1.13 (0.87–1.47) -vs DPP4i = 1.07 (0.77–1.49) Liver decompensation -*vs non GLP1-RAs = 0.81 (0.7–0.94)* -*vs DPP4i = 0.74 (0.67–0.82)* -*vs SU = 0.75 (0.67–0.84)* -vs SGLT2i = 0.94 (0.83–1.06)
Dalbeni et al 2025 [43]	Meta-analysis	T2D	1,012,482	-GLP1-RAs -Controls: non-GLP1-RAs	0.4–5.3 years	*HCC = 0.60 (0.41–0.88)*	HCC (all patients): -*vs insulin = 0.29 (0.13–0.67)* -vs oral agents = 0.81 (0.63–1.05) -vs usual care = 0.68 (0.42–1.12) HCC (non-cirrhotic patients): -*vs non-GLP1-RAs = 0.41 (0.29–0.58)* HCC (MASLD non-cirrhotic patients) -vs non-GLP1-RAs = 0.93 (0.76–1.13) HCC (cirrhotic patients): -vs non GLP1-RAs = 0.65 (0.39–1.10)

Italicized outcomes indicate primary or secondary outcomes that reached statistical significance (*p* < 0.05)

^a^Hepatic decompensation events were intented as the occurrence of ascites, spontaneous bacterial peritonitis, hepatic encephalopathy and esophageal varices;

^b^Cirrhosis complications were intented as the occurrence of cirrhosis decompensation, HCC or liver transplant, and all-cause mortality;

^c^Non-liver adverse event was intented as the occurrence of CVD, CKD, or non-liver cancer;

^d^MALO were intented as the occurrence of either decompensated cirrhosis events, HCC, or liver transplantation;

^e^Cirrhosis decompensation was intented as the occurrence of included spontaneous bacterial peritonitis, ascites onset, variceal bleeding, and hepatic encephalopathy;

^f^MALO were intented as the occurrence of either decompensated cirrhosis events, HCC, liver transplantation or MALO-related death;

^g^Hepatic decompensation events comprising ascites, esophageal varices with bleeding, hepatic failure or liver transplant;

^h^Hepatic decompensation was intented as the occurrence of hepatic encephalopathy, ascites, and variceal bleeding;

^i^MALO were intended as the occurrence of either hepatic decompensation, portal hypertension, HCC or liver transplantation;

^l^Hepatic decompensation was intented as the occurrence of ascites, hepatic encephalopathy, hepatorenal syndrome, spontaneous bacterial peritonitis, variceal bleeding;

^m^Decompensated cirrhosis was defined as liver cirrhosis, esophageal varices with or without bleeding, hepatorenal syndrome, portal hypertension, liver failure, and liver transplantation;

^n^MALO were intented as the occurrence of either decompensation of cirrhosis, hepatic failure, HCC or liver-related death;

^o^Liver-related events, defined as a composite of cirrhosis, HCC, liver decompensation (ascites, hepatic encephalopathy, variceal bleeding, spontaneous bacterial peritonitis, and hepatorenal syndrome), liver failure, liver transplantation, or liver-related death

T*2D*   type 2 diabetes, *MASLD*   metabolic dysfunction-associated steatotic liver disease, *MASH*  metabolic dysfunction-associated steatohepatitis, *GLP-1 RAs*   glucagon-like peptide-1 receptor agonist, *DPP4-i*   dipeptidyl peptidase-4 inhibitors, *SGLT2-i*   sodium–glucose cotransporter-2 inhibitors, *SUs*   sulfonylureas, *TZDs*   thiazolidinediones, *HCC * hepatocellular carcinoma, *NAFLD* nonalcholic fatty liver disease, *NASH*   non-alcoholic steatohepatitis, *CLD*  chronic liver disease, *CKD*   chronic kidney disease, *CVD*  cardiovascular disease, *HE*  hepatic encephalopathy, *MALO*   major adverse liver outcomes, *SBP*   spontaneous bacterial peritonitis, *SUs*   sulfunylureas, *T2D*   type-2 diabetes mellitus, *TZDs *  thiazolidinediones

In a large analysis of U.S. electronic health records involving approximately 30,000 patients, GLP-1RA therapy was associated with a reduced incidence of hepatic decompensation, including ascites, spontaneous bacterial peritonitis, hepatic encephalopathy and appearance or bleeding of esophageal varices, compared with most other glucose-lowering agents [30]. Importantly, the magnitude of benefit appeared to diminish in patients with established cirrhosis, suggesting a stage-dependent effect. Similarly, in a nationwide Veterans Administration cohort, initiation of GLP-1RA therapy in patients with T2D and MASLD was associated with a lower risk of progression to cirrhosis and all-cause mortality compared with DPP-4 inhibitors. Again, the protective effect was evident among non-cirrhotic individuals at baseline and lost in those with pre-existing cirrhosis [31]. Additionally, Mao et al., in a large-scale target trial emulation study, compared the use of GLP-1RA with DPP4-i observing a significant reduction in the risk of cirrhosis, as well as lower rates of cardiovascular disease, chronic kidney disease, and malignancies [32]. The study was conducted in a claims-based population of over 5 million individuals, from which 103,626 patients with T2D and MASLD initiating GLP-1RA or DPP4-i were selected. Notably, patients with other causes of chronic liver disease and advanced comorbid conditions were excluded. Interestingly, a new-user drugs head-to-head design was adopted, with inverse probability of treatment weighting to balance baseline characteristics. Patients were followed for up to 2 years under an intention-to-treat framework, with complementary per-protocol and sensitivity analyses performed to ensure the robustness of the findings [32]. In line with these observations, a head-to-head, propensity score–matched analysis demonstrated that GLP-1RAs, compared with SGLT2 inhibitors, were associated with a lower incidence of a composite outcome including hepatic decompensation, HCC, liver transplantation, and all-cause mortality (HR 0.84, 95% CI 0.73–0.97), primarily driven by fewer decompensation events, particularly ascites-related complications [33]. These findings are further strengthened by a meta-analysis of observational cohorts, in which a subgroup evaluation focusing on patients with MASLD confirmed a significantly lower incidence of cirrhotic decompensation among GLP-1RA users compared with other antidiabetic agents (HR 0.79, 95% CI 0.66–0.94) [34].

Other real-world studies have yielded conflicting results. In a Swedish register-based target trial emulation [35], while adherent GLP-1RA users with chronic liver disease and T2D had a lower risk of MALO compared with non-users, when the subgroup with MASLD was analyzed, this association was not confirmed. Notably, this subanalysis was performed exclusively under an intention-to-treat framework, which may have limited the ability to detect a treatment-specific effect in this population. Similarly, in a Korean nationwide cohort study by Bea et al., a head-to-head comparison between GLP-1RAs and SGLT2 inhibitors in patients with MASLD and T2D did not demonstrate superiority of either class with respect to hepatic decompensation events. Using a propensity score-matched design, patients initiating either treatment were followed under an as-treated framework for a mean duration of approximately 2 years. Importantly, the cohort was carefully selected to exclude other causes of chronic liver disease, and the observation period was restricted to the timeframe in which all drug classes were concurrently available, minimizing potential temporal biases. The primary outcome was a composite of hepatic decompensation events, including ascites, variceal bleeding, hepatic failure, and liver transplantation, identified through validated diagnostic and procedural codes. Over follow-up, no significant difference was observed between the two treatment groups (HR 0.93, 95% CI 0.76–1.14) [36].

When focusing on patients with established MASH cirrhosis, the results become less defined. Addition of GLP-1RAs to metformin did not confer protection against hepatic decompensation, but was associated with a survival benefit [37]. In contrast, Elsaid et al. reported that GLP-1RA therapy in patients with MASH cirrhosis was associated with a lower risk of hepatic decompensation, portal hypertension-related complications, and liver transplantation [38].

Collectively, some data from real-world studies support the results provided by clinical trials, suggesting that the use of GLP-1RAs may slow down the progression of MASH limiting the appearance of MALOs. However, other reports were not in agreement with these observations, and the benefits of GLP-1RAs in advanced liver disease are less consistent. Taken together, these results warrant the completion of prospective, dedicated studies analyzing the effects of this class of drugs on liver-specific outcomes.

## GLP-1RAs and hepatocellular carcinoma

The potential role of GLP-1RAs in modifying the risk of hepatocellular carcinoma (HCC) is attracting considerable attention. Most of the available evidence derives from the same studies where decompensation events were recorded, as outlined in the previous paragraphs (Table 1 and Fig. 1). Also in the case of HCC, differences in study design, comparator groups, patient populations (non-cirrhotic vs cirrhotic, MASLD vs mixed etiologies), and treatment exposure complicate the interpretation, underscoring the need for a critical appraisal of the available data.

### Evidence in non-cirrhotic populations with T2D and MASLD

In a target trial emulation study using U.S. insurance claim data [32], the use of GLP-1RA (vs DPP4-i) was associated with an approximately 50% reduction in HCC incidence (HR ≈ 0.53, Table 1), along with a lower risk of hepatic decompensation. Wang et al. reported reduced HCC rates among GLP-1RA users relative to patients treated with insulin [30]. However, no significant differences were observed when GLP-1RA were compared to other antidiabetic agents. Of note, the strongest protective effect (HR 0.18) was found in patients without cirrhosis at baseline, supporting the notion that early metabolic intervention may more effectively interrupt the chain of events leading to the appearance of HCC. In keeping with these observations, a markedly reduced risk of incident HCC and cirrhosis was reported in individuals with T2D treated with GLP-1RAs vs. those treated with long-acting insulin, after propensity score matching [39]. In contrast, in the study by Kanwal et al. [31], GLP-1RA therapy, compared to DPP4-i, did not significantly decrease the incidence of HCC. This observation is at difference with the significant reduction in all-cause mortality mentioned above. It should be considered that HCC incidence was not a predefined primary endpoint in this analysis, and this likely limited the statistical power to detect a significant difference. A lack of protection from HCC was also reported in the Swedish population-based cohort study [35], where the comparator group pooled all antidiabetic regimens and included heterogeneous etiologies (e.g., viral and alcohol-related liver disease), potentially diluting class-specific effects on MASLD-related HCC. Similarly, in a recent cohort study involving approximately 30,000 patients with MASLD and T2D, while therapy with GLP-1RA, compared with SGLT2 inhibitors, was associated with a lower incidence of MALO, it did not show any superiority in reducing the incidence of HCC [33]. Although patients with established cirrhosis were included in this study, their proportion (roughly 4%) is unlikely to have significantly influenced the observed effect. More plausibly, the lack of superiority may reflect the metabolic efficacy of the SGLT2 inhibitors, which also reduce weight and hyperinsulinemia, with effects on hepatocarcinogenesis potentially similar to the ones of GLP-1RAs. A similar interpretation may apply to the findings reported by Bea et al. [36], where no superiority of GLP-1RAs over SGLT2 inhibitors was observed in terms of HCC prevention, evaluated as a secondary endpoint. In line with these latter findings, a recent study, conducted in a well-characterized cohort of patients with T2D and MASLD after propensity score matching, did not demonstrate any significant differences between GLP-1RAs and either SGLT2 inhibitors or DPP-4 inhibitors in terms of incident HCC or cirrhosis, whether assessed as composite or individual endpoints [40]. It should be noted, however, that the median age of the study population was relatively low (54 years, IQR 47–60), the duration of follow-up was among the shortest reported in non-cirrhotic cohorts, and the exclusion criteria were particularly strict (e.g., exclusion of smokers), potentially introducing a selection bias. These factors likely contributed to the low number of events observed, which in some comparisons may have limited the statistical power to detect meaningful differences [40].

Collectively, while some studies demonstrate a possible chemopreventive effect of GLP-1RAs against HCC, this was not unequivocally demonstrated in all studies. Interestingly, the limiting effects of GLP-1RAs on the development of HCC are likely linked to their potent metabolic actions, since they are shared by SGLT2 inhibitors, which also induce weight loss and improvement in insulin resistance.

### Evidence in patients with MASH cirrhosis

The impact of GLP-1RAs on HCC prevention in individuals with T2D and MASH who have already progressed to cirrhosis is also controversial. In a multicenter study, GLP-1RA therapy, compared with all other antidiabetic regimens, was associated with a significantly lower risk of HCC (adjusted HR for HCC ≈ 0.37) [38]. Similarly, in a subgroup analysis that included 6588 patients with T2D and MASH cirrhosis, Wang et al. reported a protective association of GLP-1RAs, although this effect was evident only when compared with insulin-based regimens [30]. Thus, also in this case, the superiority of GLP-1RAs was limited to the comparison with an agent that does not impact the metabolic profile of the patient. In a group of patients with cirrhosis of all causes and T2D, the combination of GLP-1RAs with metformin was associated with lower incidence of HCC compared with metformin monotherapy [33]. However, when the MASH cirrhosis subgroup was analyzed, only the survival benefit remained statistically significant. Accordingly, Kanwal et al. observed no significant reduction in HCC incidence among patients with MASH cirrhosis at baseline [31]. Likewise, in a subgroup of compensated all-cause cirrhosis, no benefit on MALO, including HCC, was observed in patients receiving GLP-1RA therapy [35].

The discrepancies across the above studies should be interpreted in the light of inherent limitations linked to observational datasets. Differences in primary endpoints (with HCC often assessed as a secondary or exploratory outcome), GLP-1RA type and dosing, observation periods, treatment discontinuation rates, comparator agents and their dosages, as well as concomitant therapies, may all have contributed to heterogeneity in effect estimates, and complicated direct comparisons. Overall, the available evidence suggests that also in the setting of advanced compensated liver disease, there are signals of a possible preventive effect of GLP-1RAs on HCC development, but additional, prospective studies should be designed to specifically evaluate this outcome.

### Evidence from meta-analyses

**Fig. 2:**
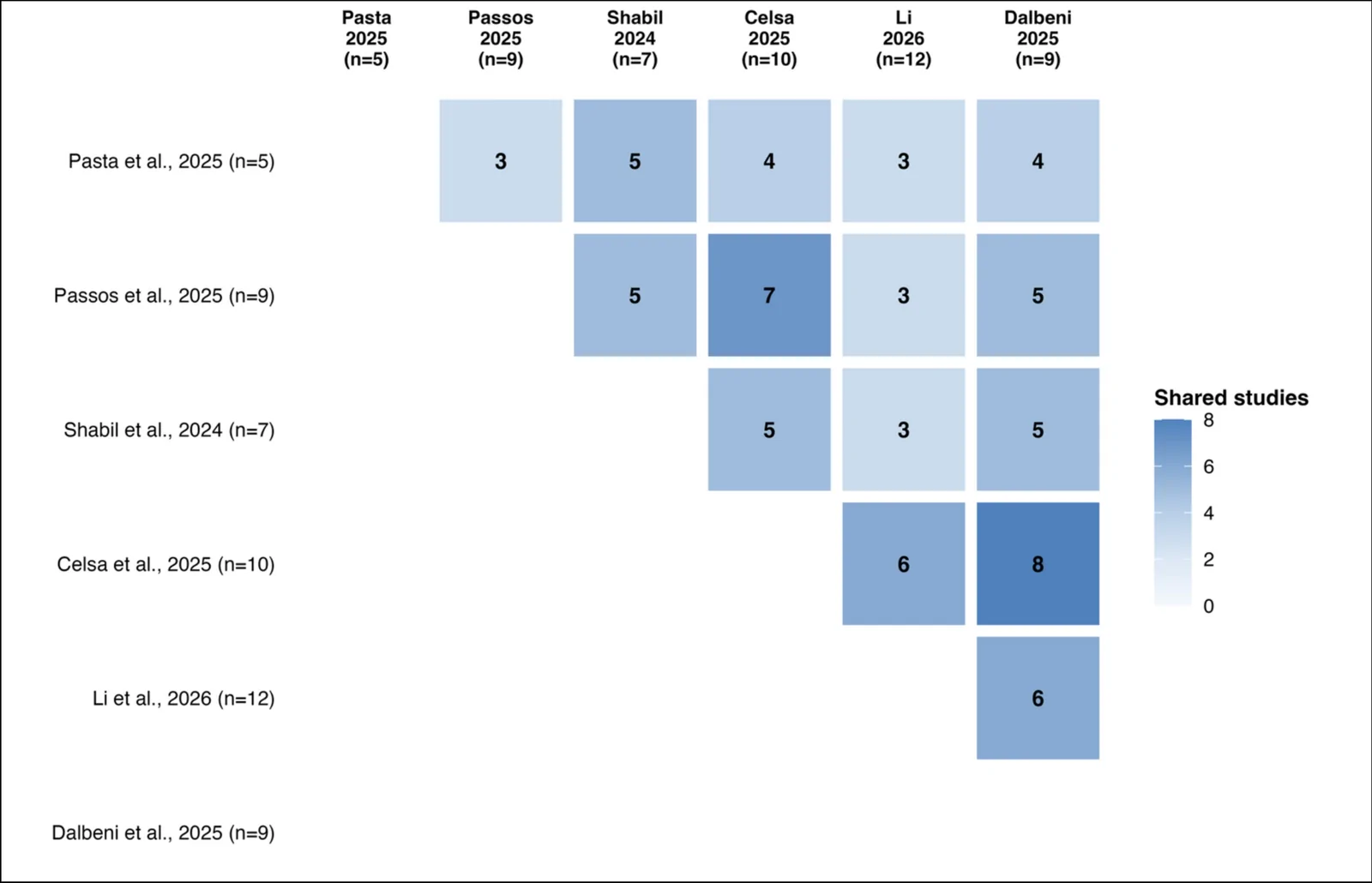
Pairwise overlap of primary studies across meta-analyses. Triangular heatmap showing the pairwise overlap in primary studies among the meta-analyses included in this review. Each cell reports the number of studies shared by the corresponding pair of meta-analyses, with darker shading indicating greater overlap. Only unique pairwise comparisons are displayed, whereas diagonal cells and mirrored duplicates are omitted. The number of primary studies included in each meta-analysis is reported alongside the corresponding study label. This figure provides a direct estimate of the extent to which different evidence syntheses are based on a shared underlying set of primary studies

Several systematic reviews and meta-analyses have synthesized the growing body of observational evidence on GLP-1RA therapy and the incidence of hepatocellular carcinoma in patients with T2D, including subsets with documented metabolic liver disease (Table 1 and Fig. 2). In a recent meta-analysis of pooled comparative cohorts of patients with T2D, the use of GLP-1RA was associated with a significantly lower risk of incident HCC (HR 0.42), with similar results in a subgroup of patients with cirrhosis (HR 0.44, Table 1) [16]. However, two of the largest contributing studies [39, 41] included only a very limited proportion of patients with confirmed MASLD/MASH (approximately 2% and 1.2%, respectively). Moreover, this meta-analysis did not substratify the non-GLP-1RA users according to the alternative drugs used. Similarly, Shabil et al. reported a pooled HR of 0.41, suggesting a nearly 60% reduction in HCC risk among T2D patients using GLP-1RA [42]. Analyzing the comparison with other antidiabetic medications, GLP-1RAs were superior against no use of GLP-1RA (HR 0.47) as a whole, while in subgroup analyses involving other antidiabetic therapies, GLP-1RAs were superior only to insulin (HR 0.36) or sulfonylureas. This meta-analysis also included studies with either very low representation of MASLD patients [39, 41] or without clear etiologic stratification [43]. A very recent meta-analysis by Dalbeni et al. broadly yielded similar results, showing a 40% reduction in the risk of HCC appearance among patients receiving GLP-1RAs compared with non-users. Also in that study, subgroup analyses suggested that the overall effect was largely driven by comparisons against insulin and by a predominance of benefit in non-cirrhotic populations. Moreover, when the analysis was restricted to patients specifically with MASLD, statistical significance was lost despite a persistent trend in favor of GLP-1RAs [44].

Celsa et al. conducted one of the largest systematic reviews to date, including over 1.4 million individuals with T2D. GLP-1RA therapy was associated with a reduced risk of major adverse liver outcomes, a composite including cirrhotic decompensation, hepatic failure, HCC, or liver-related death [45]. However, when the individual endpoints were analyzed separately, only the reduction of decompensation was significant, while for HCC, the pooled effect estimate did not reach statistical significance. Notably, effect sizes varied significantly across comparators, with a clearer protective signal observed again against insulin and sulfonylureas, while differences were markedly attenuated versus SGLT2 inhibitors, metformin, or DPP4 inhibitors. As with previously discussed meta-analyses, the inclusion of cohorts with variable proportions of MASLD patients, differing percentages of cirrhotic patients, heterogeneous comparators, and variable dosing regimens are likely to have contributed to between-study variability. Finally, in a recent meta-analysis specifically designed for patients with T2D and MASLD [46], GLP-1RAs were superior to non-GLP-1RA therapies in reducing liver-related events, and this finding remained consistent even after full covariate adjustment or restricting the analysis to studies with non-overlapping databases. Here again, however, the observed efficacy appeared to be driven mainly by hepatic decompensation and by comparisons against insulin, DPP4 inhibitors, and sulfonylureas, rather than by a robust and consistent reduction in HCC across all the treatments compared.

Collectively, meta-analytic data reinforce the overall signal of hepatoprotection associated with GLP-1RAs. However, the validity of these pooled estimates is limited by between-study variability in disease etiology, cirrhosis status, comparator selection, treatment exposure, and outcome hierarchy. As such, the existing meta-analyses ultimately highlight the need for dedicated prospective studies specifically designed to assess HCC prevention in well-characterized MASLD populations.

### Evidence in obesity-related MASLD without T2D

The increasing use of GLP-1RAs for obesity management in individuals without diabetes raises the question of whether these agents may also modify the risk of HCC in obesity-related MASLD. However, specific studies evaluating incident HCC in non-diabetic individuals treated with GLP-1RAs are lacking. Therefore, any potential chemopreventive effect in this population remains hypothetical, and is mainly based on data on weight reduction, liver fat content, MASH resolution, and fibrosis-related endpoints as emerging from clinical trials investigating GLP-1RAs in MASH [19, 26, 27].

Epidemiological data suggest that obesity and T2D do not equally contribute to MASLD-related HCC risk. In a recent meta-analysis of modifiable risk factors in MASLD, overweight/obesity was associated with a moderate increase in incident HCC, whereas the association was stronger in diabetes [47]. Similarly, a systematic review and meta-analysis including more than 700,000 patients with MASLD confirmed a greater contribution of diabetes than obesity alone to HCC development, with pooled risk estimates of OR 2.38 and 1.46, respectively [48]. Indeed, the relationship between obesity and HCC appears to be strongly influenced by the metabolic health status [49]. Consistently, prospective studies indicate that the risk of HCC increases with the number of metabolic comorbidities, including diabetes, hypertension, and dyslipidemia [50]. Dedicated prospective studies with adequate duration, large sample size, careful characterization of MASLD severity, and stratification by glycemic status are needed to determine whether the histological and metabolic benefits of GLP-1RAs translate into a measurable reduction in HCC incidence in individuals with non-diabetic obesity.

### Comparisons with other glucose-lowering therapies

The therapeutic management of patients with T2D and MASLD encompasses a broad spectrum of glucose-lowering agents, each exerting distinct metabolic effects that may influence hepatocarcinogenesis. In this context, head-to-head comparisons are critical to determine whether the potential chemopreventive effect of GLP-1RAs reflects intrinsic hepatoprotective properties or relative advantages over agents that promote weight gain, hyperinsulinemia, or lipotoxic stress.

### GLP-1RAs versus insulin and sulfonylureas

The most pronounced reductions in HCC incidence have been observed when GLP-1RAs were compared with insulin or sulfonylureas. Multiple cohort studies and meta-analyses [30, 39, 42, 45] reported hazard ratios in the range of 0.3–0.5 for HCC among GLP-1RA users relative to insulin-treated patients, indicating a substantially lower carcinogenic risk. These findings likely reflect the deleterious hepatic effects of chronic exogenous hyperinsulinemia and weight-promoting therapies, which enhance insulin/IGF-1 signaling, lipogenesis, and inflammation. A comparable advantage was seen over sulfonylureas [30], further supporting the hypothesis that the chemopreventive potential of GLP-1RAs largely stems from mitigation of tumorigenic mechanisms driven by hyperinsulinemia.

### GLP-1RAs versus metformin and DPP-4 inhibitors

Comparisons against metformin have yielded more attenuated differences. Metformin has been shown to exert hepatoprotective and antineoplastic effects through AMPK activation, suppression of mTOR signaling, and reduction of hepatic steatosis [51]. GLP-1RAs demonstrated only incremental risk reductions when compared directly with metformin. However, evidence from combination therapy suggests additive benefits beyond metformin alone, suggesting partially distinct and complementary mechanisms of action [30, 37]. DPP-4 inhibitors exhibit more modest metabolic and hepatic effects. While they improve glycemic control, their impact on body weight, insulin resistance, and hepatic inflammation appears limited compared with GLP-1RAs. Accordingly, across multiple observational studies, DPP-4i consistently underperformed relative to GLP-1RAs in terms of cirrhosis progression [31], hepatic decompensation [30], and HCC incidence [32].

### GLP-1RAs versus SGLT2 inhibitors

The comparison between GLP-1RAs and SGLT2i is particularly relevant given their widespread use and accumulating evidence suggesting that both classes exert potential liver-related benefits in patients with type 2 diabetes and MASLD [52–54]. However, data from head-to-head observational studies remain heterogeneous and, at times, conflicting. In a predominantly non-cirrhotic population with T2D and MASLD, Kuo et al. reported a significantly lower risk of MALOs with GLP-1RAs compared with SGLT2 inhibitors (HR 0.84), an effect mainly driven by a reduction in hepatic decompensation, whereas no meaningful difference was observed in HCC incidence [33]. Along the same line, in a subgroup analysis, GLP-1RAs were associated with a significant reduction in hepatic decompensation compared with SGLT2 inhibitors, although this analysis was conducted in a broader diabetic population, without specific restriction according to the presence or absence of liver disease or its etiology [34]. Similarly, in a well-designed propensity score-matched study with rigorous inclusion criteria, a transient benefit favoring GLP-1RAs, particularly for hepatic decompensation, was observed, although this difference progressively attenuated and lost statistical significance by 2 years [40]. Consistent with this pattern, Celsa et al., in a subgroup analysis, showed a lower incidence of composite MALOs with GLP-1RAs compared with SGLT2 inhibitors, whereas no significant difference was observed when the analysis was restricted to hepatic decompensation alone [45]. In contrast, Bea et al. found no significant difference between SGLT2 inhibitors and GLP-1RAs for hepatic decompensation or HCC in the overall analysis [36]. Notably, subgroup and secondary analyses suggested a more favorable effect of SGLT2 inhibitors in patients younger than 65 years and in those with pre-existing cirrhosis, although the latter subgroup was relatively small. Conversely, GLP-1RAs appeared to perform better with respect to liver transplantation and, to a lesser extent, hepatic failure. These findings should be interpreted in light of the exclusively Asian ethnicity of this cohort. Moreover, a recent meta-analysis did not show any significant differences between SGLT2 inhibitors and GLP-1RAs for liver-related events or for HCC specifically [46]. When the analysis was restricted to the non-Asian population, a significant advantage of GLP-1RAs for liver-related events was observed. Taken together, these apparently discordant findings highlight the limited reliability of subgroup analyses in identifying robust treatment trajectories outside the primary study design, especially when effect sizes are modest and endpoints are heterogeneous.

### GLP-1RAs versus bariatric surgery

When compared with bariatric surgery, GLP-1RA therapy generally yields smaller effects on weight loss, insulin resistance, and histological resolution of steatohepatitis [55–58] which may translate into a comparatively lower magnitude of protection against cirrhosis progression and HCC. In a recent multinational cohort study including over 180,000 patients with severe obesity (BMI ≥ 35 kg/m^2^) [59], bariatric surgery was associated with lower incidence of new-onset MASH (HR 0.51) and HCC (HR 0.30) compared with GLP-1RA therapy (liraglutide or semaglutide). However, these differences were no longer significant in the subgroup with type 2 diabetes, in which risk estimates were neutral (HR ≈ 1.1 for MASH), and even trended toward a lower risk of cirrhosis among GLP-1RA users. Moreover, the study population was not recruited for MASLD, included only about one-third of patients with metabolic comorbidities, and analyses were not adjusted for GLP-1RA dosage or treatment duration, making these results poorly generalizable to the broader MASLD population. These findings allow to hypothesize that while bariatric surgery may confer stronger benefits in metabolically healthy obesity, GLP-1RAs could provide comparable hepatic protection in diabetic and metabolically dysregulated patients, particularly when high-dose or long-acting agents are used.

### Future perspectives: next-generation incretin and combination metabolic therapies

Emerging therapeutic strategies for MASH are increasingly moving from single-pathway interventions toward multi-target metabolic approaches. Among conventional add-on regimens, the association between GLP-1RAs and SGLT2 inhibitors is particularly attractive, as the two drug classes exert complementary effects on body weight, glycemic control, insulin resistance, blood pressure, and cardio-renal risk, all of which are closely linked to MASLD progression. Recent real-world studies in patients with MASLD and T2D suggest that combination therapy with GLP-1RA/SGLT2i may be associated with a lower risk of major adverse liver outcomes and reduced fibrosis progression compared with monotherapy [60, 61]. However, these data did not include a specific assessment of HCC prevention.

A second strategy may be represented by the combination of GLP-1-based therapies with liver-directed agents. In a secondary analysis of the MAESTRO-NASH trial, background treatment with GLP-1RAs did not attenuate the histological efficacy of resmetirom, and weight loss ≥ 5% was associated with greater improvements in MASH-related outcomes [62]. These findings support the compatibility of systemic metabolic correction and liver-directed therapy, although they did not prove pharmacological synergy. Similarly, the addition of efruxifermin, a FGF21 analog, to stable GLP-1RA therapy in patients with T2D and MASH showed favorable short-term effects on hepatic fat and non-invasive markers of liver injury and fibrosis, supporting the rationale for incretin–FGF21 combination strategies [63].

At present, the strongest clinical proof-of-concept for multi-target metabolic therapy comes from unimolecular co-agonists such as tirzepatide, a dual GIP/GLP-1 receptor agonist, which improved MASH resolution and fibrosis-related endpoints in patients with MASH and F2–F3 fibrosis [64]. Survodutide, a dual GLP-1/glucagon receptor agonist, significantly improved MASH without worsening of fibrosis in a phase 2 trial and also reduced liver fat content [65]. Retatrutide, a triple GIP/GLP-1/glucagon receptor agonist, produced marked reductions in liver fat in participants with MASLD in a phase 2a trial [66].

Although none of these approaches has yet been shown to reduce HCC incidence, they provide a strong rationale for future trials testing whether deeper and sustained improvements in weight, insulin resistance, steatohepatitis, and fibrosis can translate into reduced cirrhosis progression and HCC risk.

## Pathophysiological mechanisms underlying GLP-1RA-mediated protection against hepatocellular carcinoma

Although epidemiological and clinical evidence increasingly support a protective association between GLP-1RA therapy and the risk of HCC, key questions concerning the biological mechanisms underlying this effect have yet to be fully addressed. Over the past decade, translational research has identified multiple interconnected pathways through which GLP-1RAs may confer hepatoprotective, anti-inflammatory, and antitumor effects. These actions extend well beyond glycemic regulation, encompassing hepatocellular metabolism, modulation of immune cell biology, fibrogenic pathways, regulation of bile acid metabolism and the gut–liver axis, and even epigenetic remodeling [21, 22]. Understanding these mechanisms may provide a crucial link between the observed clinical associations and molecular mechanisms, reinforcing the rationale for GLP-1RAs as potential disease-modifying and chemopreventive agents against HCC.

**Fig. 3:**
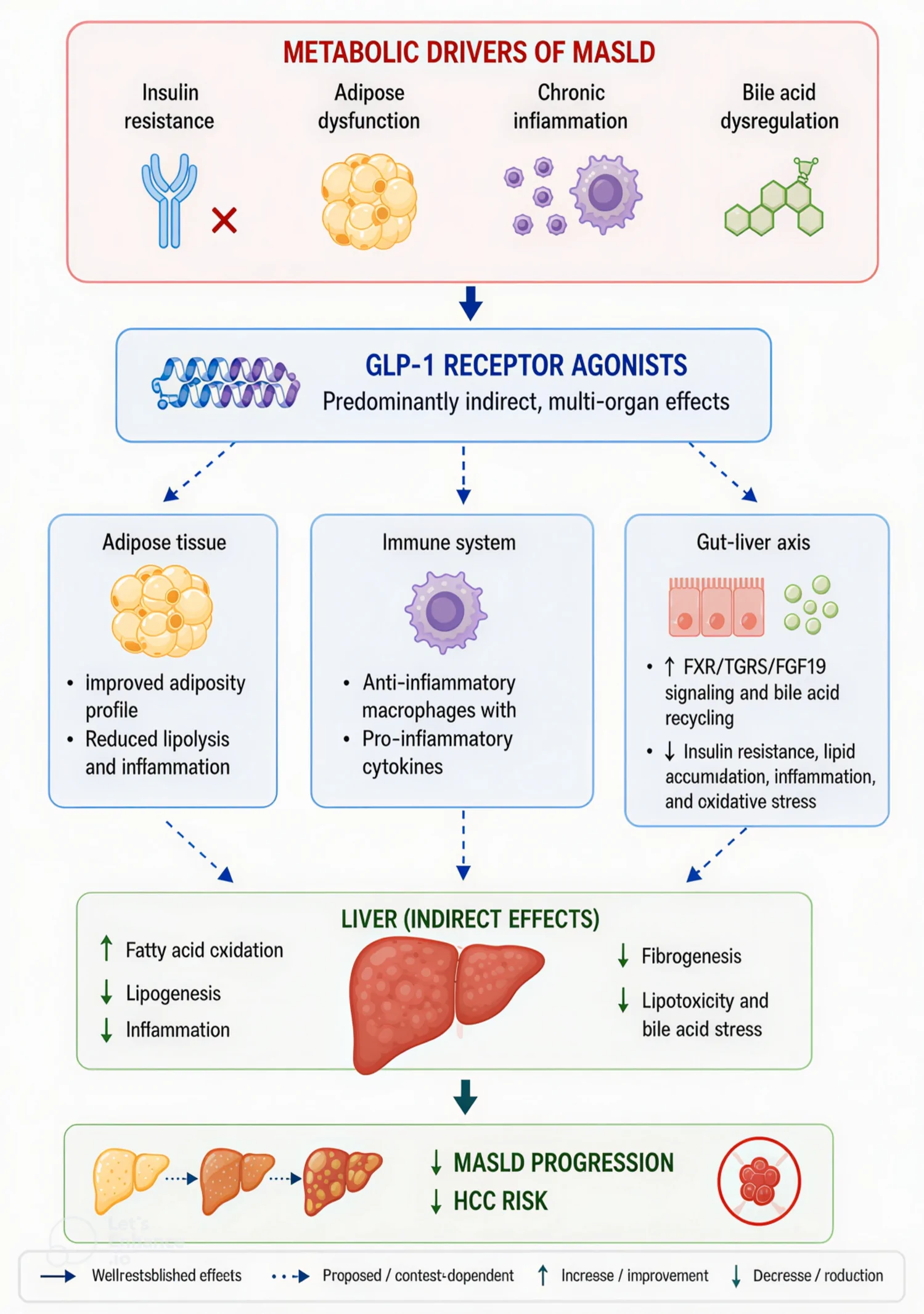
Figure 3. Indirect multi-organ mechanisms of GLP-1 receptor agonist–mediated hepatoprotection in MASLD. Metabolic dysfunction in MASLD is sustained by insulin resistance, adipose-tissue dysfunction, chronic inflammation, and bile acid dysregulation. Because functional GLP-1 receptor expression in human hepatocytes appears limited, the hepatic effects of GLP-1 receptor agonists are thought to occur predominantly through indirect, multi-organ mechanisms. GLP-1 receptor agonists improve adipose-tissue function, reduce lipolysis and inflammatory signaling, modulate immune-cell activation, and influence the gut–liver axis, including bile acid recycling and FXR/TGR5/FGF19-related pathways. These systemic actions converge on the liver to reduce steatosis, lipogenesis, lipotoxicity, inflammation, oxidative and bile acid stress, and fibrogenic remodeling, thereby potentially slowing MASLD progression and lowering the permissive substrate for hepatocarcinogenesis. Solid arrows indicate well-established effects; dashed arrows indicate proposed or context-dependent mechanisms

The mechanisms underlying the beneficial effects of GLP-1RA on metabolic liver diseases such as MASLD have been recently reviewed elsewhere [67]. The liver was initially considered a potential direct target of GLP-1 based on early in vitro studies reporting GLP-1R expression in human hepatocytes and hepatoma cell lines, as well as GLP-1-dependent modulation of lipid metabolism and insulin signaling [68–70]. However, accumulating evidence from more recent and methodologically rigorous studies has substantially revised this paradigm. Comprehensive analyses of GLP-1R distribution using validated molecular tools, including receptor-specific approaches and single-cell transcriptomic data, indicate that functional GLP-1R expression in primary human hepatocytes is negligible or absent in vivo [21, 22, 71]. Recently, it has been confirmed that even most of non-parenchymal cells within the liver lack expression of GLP-1 receptors [72]. As a result, the signaling mechanisms inferred from earlier in vitro models are now widely interpreted as model-specific phenomena, potentially influenced by hepatoma-derived cell lines, long-term culture conditions, low-abundance transcript detection, or contamination with non-parenchymal GLP-1R-expressing cells. Within this updated framework, the biologic actions previously attributed to direct hepatocellular GLP-1R activation are more plausibly explained as downstream consequences of indirect mechanisms, involving multiple cell types (Fig. 3).

### Anti-inflammatory and immunomodulatory actions of GLP-1RA relevant to the pathophysiology of MASLD

Accumulating evidence indicates that the anti-inflammatory effects of GLP-1RAs in MASLD are largely mediated through indirect and multicellular mechanisms rather than direct hepatocyte-autonomous signaling. Inhibition of NF-κB and NLRP3 inflammasome activation by GLP-1R signaling has been demonstrated in metabolically active tissues such as adipocytes and immune cells [73]. This results in reduced secretion of pro-inflammatory cytokines including IL-1β and TNF-α, and these effects are now interpreted as upstream modulators of hepatic inflammation.

Within the liver, GLP-1R expression has been detected in non-parenchymal cells, particularly macrophages and hepatic stellate cells, which play a central role in orchestrating inflammation and fibrogenesis. In macrophages, GLP-1R activation modulates immune polarization, promoting an anti-inflammatory M2 phenotype and attenuating tissue injury in models of hepatic ischemia–reperfusion and metabolic stress [74]. This shift in macrophage phenotype reduces the inflammatory burden and cytokine-driven hepatocyte injury that characterizes steatohepatitis. Similarly, in hepatic stellate cells, GLP-1RAs have been reported to reduce lipotoxic stress and suppress the expression of key pro-fibrogenic markers, including COL1A1 and α-smooth muscle actin (α-SMA), while downregulating inflammatory mediators involved in extracellular matrix deposition and fibrosis progression [75]. Through these actions on non-parenchymal cell populations, GLP-1RAs could indirectly dampen hepatic inflammation, limit fibrogenic remodeling, and limit the establishment of a pro-oncogenic microenvironment within the liver. However, recent data failed to confirm the presence of GLP-1R also in non-parenchymal liver cells, both in mice and in humans [72], and the final relevance of these mechanisms remains uncertain.

### In vivo evidence linking GLP-1RAs to hepatocellular carcinoma development

In vivo studies using metabolic, steatohepatitis-associated, and liver cancer-prone murine models support a role for GLP-1RAs in modulating HCC development and progression. Although many experimental systems were originally designed to model obesity or steatohepatitis rather than liver cancer per se, they consistently interrogate biological pathways—such as hyperinsulinemia, chronic inflammation, oxidative stress, and hepatocyte apoptosis—that are central to hepatocarcinogenesis in metabolic liver disease.

In diet-induced obese and high-fat-fed mice, GLP-1 gene therapy or pharmacological GLP-1RA treatment reduced body weight, hepatic steatosis, and insulin resistance while simultaneously suppressing systemic inflammation and hyperinsulinemia [76]. These systemic effects are highly relevant to HCC prevention, as sustained insulin/IGF-1 signaling and inflammatory stress are key drivers of hepatocyte proliferation, genomic instability, and malignant transformation. More direct links between GLP-1RA treatment and suppression of carcinogenic pathways emerge from models that combine steatohepatitis with a tumor-permissive hepatic microenvironment. In the Gubra Amylin Nash (GAN) diet-induced MASH model, semaglutide not only improved steatohepatitis and fibrosis but also reduced hepatocyte apoptosis, oxidative stress, and markers of cellular injury and proliferation [77]. These features are recognized hallmarks of the pre-neoplastic liver and are strongly implicated in the transition from chronic liver damage to HCC.

Other studies have provided more direct evidence of antitumor activity in murine models of diet-induced liver cancer. In a high-fat-diet-induced HCC model, long-term semaglutide administration significantly reduced hepatic tumor burden and downregulated oncogenic signaling pathways involved in lipid metabolism, inflammation, and cell proliferation [78]. Proteomic analyses further revealed attenuation of oxidative stress responses and restoration of mitochondrial homeostasis, mechanisms directly linked to reduced tumor initiation and slower tumor progression. Proteomic analyses of semaglutide-treated obese mice confirmed downregulation of oncogenic pathways involved in cell proliferation, inflammation, and lipid metabolism [78]. In diethylnitrosamine-treated mice consuming control or high-fat high-carbohydrate diet, administration of exendin-4 reduced HCC multiplicity in obese DEN-treated mice. Tumor suppression was associated with activation of cyclic AMP (cAMP) and protein kinase A (PKA), and with downregulation of epidermal growth factor receptor and STAT3 [79]. Moreover, in a diabetic murine MASH-HCC model, GLP-1R agonist treatment prevented tumor progression and reduced hepatic cytokine expression [80]. Finally, in a subcutaneous model of HCC, liraglutide improved the antitumor immune responses, an effect mediated by natural killer cells but not CD8 + T cells. Additionally, liraglutide suppressed the IL-6/STAT3 signaling pathway in HCC cells [81].

These results in animal models are supported by studies in cultured cells, although some of them have been conducted in hepatoblastoma HepG2 cells. Krause et al. [82] reported that liraglutide induces autophagy and senescence through increased expression of TGF-β1. In HuH7 cells, GLP-1, but not GIP, reduced cell migration induced by soluble mediator, and attenuated the phosphorylation of stress-activated protein kinase/*c-Jun* N-terminal kinase (SAPK/JNK) in a cAMP-dependent fashion [83].

Additional indirect support for reduced HCC susceptibility arises from modulation of the gut–liver axis. Studies using dual GLP-1/GLP-2 receptor agonists showed reshaping of gut microbiota composition and improvement of metabolic and inflammatory profiles in obese mice, potentially limiting hepatic exposure to pro-inflammatory and pro-carcinogenic microbial products [84]. Given the established role of intestinal dysbiosis and endotoxemia in promoting liver carcinogenesis, these findings further strengthen the biological plausibility of GLP-1-mediated protection against HCC.

Beyond intestinal dysbiosis, growing evidence indicates that microbiome-related mechanisms may contribute directly to hepatocarcinogenesis through modulation of the hepatic and tumor microenvironment. Recent studies have identified distinct microbial signatures not only in the gut but also within the liver and HCC itself, supporting the concept of an intrahepatic and intratumoral microbiome [85–87]. These microbial communities may influence local inflammation, metabolic reprogramming, and immune surveillance, thereby contributing to tumor initiation and progression. In addition, increased intestinal permeability and translocation of microbial products promote chronic inflammation and fibrogenesis, creating a permissive environment for malignant transformation [86]. Microbiome-derived metabolites, including secondary bile acids and short-chain fatty acids, also participate in the regulation of hepatic immune homeostasis and antitumor responses [88, 89]. Although direct evidence linking GLP-1RAs to modulation of the intrahepatic or intratumoral microbiome remains limited, these agents have been shown to improve gut barrier integrity, reduce endotoxemia, and favorably reshape microbial composition in experimental models [84]. Therefore, modulation of microbiome–immune interactions may represent an additional mechanism through which GLP-1RAs contribute to attenuation of the pro-oncogenic milieu associated with MASLD and HCC development.

Emerging research highlights that GLP-1RAs may exert long-term protective effects via epigenetic mechanisms. Hanna et al. [90] proposed that GLP-1R activation promotes chromatin remodeling and histone acetylation patterns consistent with hepatic rejuvenation and HCC prevention during aging. This suggests that incretin signaling may influence transcriptional programs beyond classical metabolic regulation, aligning with recent observations of improved mitochondrial function and reduced senescence markers in treated livers [78, 90].

**Fig. 4:**
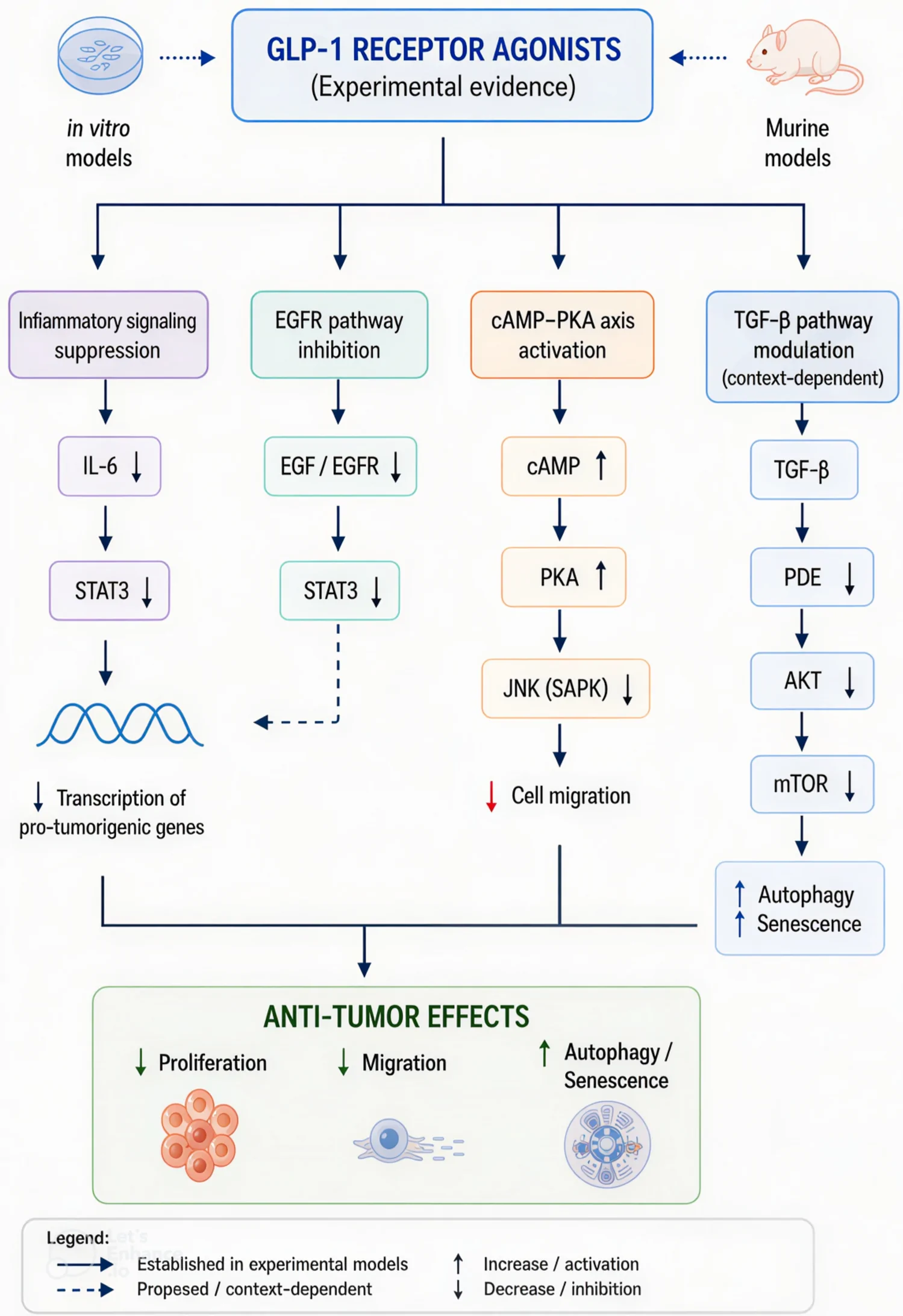
Figure 4. Antitumor signaling pathways modulated by GLP-1 receptor agonists emerging from preclinical studies. Experimental studies indicate that GLP-1 receptor agonists may interfere with hepatocarcinogenesis by suppressing IL-6/STAT3 and EGF/EGFR signaling, activating the cAMP–PKA axis with downstream inhibition of SAPK/JNK-dependent migration, and modulating TGF-β–related pathways linked to AKT/mTOR inhibition, autophagy, and senescence. These model-dependent effects converge on reduced proliferation and migration and increased autophagy/senescence, supporting a potential antitumor role of GLP1-RAin preclinical settings. Solid arrows denote experimentally supported effects; dashed arrows denote proposed or context-dependent mechanisms. *AKT* protein kinase B, *cAMP* cyclic adenosine monophosphate, *EGF* epidermal growth factor, *EGFR* epidermal growth factor receptor, *GLP-1* glucagon-like peptide-1, *IL-6* interleukin-6, *JNK* c-Jun N-terminal kinase, *mTOR* mechanistic target of rapamycin, *PDE* phosphodiesterase, *PKA* protein kinase A, *SAPK* stress-activated protein kinase, *STAT3* signal transducer and activator of transcription 3, *TGF-β* transforming growth factor-β

Collectively, these in vivo data indicate that GLP-1RAs interfere with multiple convergent pathways involved in hepatocarcinogenesis, including metabolic dysregulation, chronic inflammation, oxidative and mitochondrial stress, and tumor-promoting signaling. Rather than acting solely as anti-steatotic agents, GLP-1RAs appear to attenuate both the permissive hepatic microenvironment and, in selected models, tumor burden itself (Fig. 4).

### Crosstalk with bile acid and FXR/FGF19 signaling

Bile acid metabolism constitutes a central node linking metabolic stress and liver carcinogenesis. Intestinal GLP-1 activation and surgical models mimicking enhanced GLP-1 release, such as ileal interposition, increase bile acid recycling and protect against obesity-related liver disease by activating bile acid receptors and FGF19 signaling [91, 92].

Pharmacological modulation of the FXR/TGR5/GLP-1 pathway improves insulin resistance and lipid metabolism in MASLD mice. Pharmacological activation of intestinal FXR reshapes bile acid signaling and engages downstream TGR5-dependent incretin pathways, leading to increased GLP-1 release and significant improvements in hepatic insulin resistance, lipid accumulation, and inflammatory injury in murine models of MASLD [93].

Through these mechanisms, GLP-1RAs may reduce bile acid-induced oxidative stress and DNA damage, limiting one of the key triggers of hepatocyte transformation.

### Adipokines in liver disease and their modulation by GLP-1 receptor agonists

Adipokines secreted by adipose tissue play essential endocrine roles in hepatic metabolism, inflammation, and tumorigenesis. In MASLD, dysregulated adipokines—particularly leptin—contribute to steatosis, insulin resistance, and fibrogenesis through effects on β-oxidation, lipogenesis, and hepatic stellate cell activation [94]. Chronic hyperleptinemia, characteristic of obesity, promotes hepatic inflammation and fibrosis and is mechanistically linked to progression toward HCC through pro-proliferative, anti-apoptotic, and pro-angiogenic signaling pathways, including JAK/STAT3 and PI3K/Akt activation [95, 96]. Moreover, several novel adipokines—including Metrnl, RBP4, vaspin, and LCN-2—have recently been implicated in HCC biology, influencing tumor growth, metastasis, and prognosis, underscoring the importance of adipose–liver endocrine crosstalk in hepatocarcinogenesis [97].

Growing evidence indicates that at least part of the beneficial metabolic effects of GLP-1RAs are mediated by modulation of adipokine secretion and improvement of adipose tissue function. GLP-1RAs have been shown to correct obesity-associated hyperleptinemia and leptin resistance [98–100]. In parallel, GLP-1RAs decrease resistin concentrations and enhance insulin sensitivity, supporting an anti-inflammatory shift in adipose tissue signaling [98, 101]. Beyond leptin and resistin, incretin-based therapies such as liraglutide and exenatide also elevate adiponectin and omentin-1, adipokines with insulin-sensitizing properties, thereby contributing to improved glucose and lipid homeostasis [102, 103]. GLP-1RAs may also restore leptin responsiveness through microbial metabolites such as inosine, which activate adipose-macrophage A2A signaling [103]. Collectively, these findings support a multifaceted role of GLP-1RAs in modulating adipose endocrine function, ultimately improving metabolic health and reducing obesity-associated cardiometabolic and liver-related risk.

### Obesity-driven versus diabetes-driven MASLD-HCC: mechanistic implications for GLP-1RA-mediated protection

Although obesity and T2D frequently coexist in patients with MASLD, their contribution to hepatocarcinogenesis should not be considered biologically interchangeable. Mice receiving STZ together with a high-fat diet develop steatosis, MASH, fibrosis, and HCC, in the setting of metabolic dysfunction. In this model, HFD-only mice are less prone to develop HCC when STZ is not co-administered, suggesting that the diabetic-metabolic milieu may induce a stronger pro-carcinogenic liver phenotype [104]. This concept is consistent with earlier MASH–HCC models showing that insulin resistance may promote hepatocarcinogenesis even in the absence of cirrhosis, in a model combining CDAA with low-dose CCl_4_ injection [105]. These findings support the hypothesis that diabetes-related hepatocarcinogenesis may involve not only a greater burden of MASH and fibrosis, but also activation of insulin/IGF-related proliferative pathways. However, insulin signaling is complex and tissue-specific, and may also modulate carcinogenesis through extrahepatic mechanisms. In diabetic mice with MASH, restoration of the intestinal action of insulin reduced dysbiosis, improved antimicrobial peptide production, and reshaped the bile acid profile, ultimately suppressing hepatocarcinogenesis [106]. These data provide a framework explaining possible different effects of GLP-1RAs in diabetic and non-diabetic, obesity-related, MASLD. In non-diabetic obesity, GLP-1RA-mediated protection would be expected to derive mainly from weight loss and improvement in steatohepatitis-related injury. In obesity with T2D, the same agents may additionally modulate glycemic control, insulin resistance, insulin/IGF-related signaling, and diabetes-associated alterations in the gut–liver axis.

### Integrative model of GLP-1RA-mediated hepatoprotection

Collectively, in vitro and in vivo findings converge on a model where GLP-1RAs modulate multiple interconnected pathways, as they: (1) enhance insulin sensitivity and fatty acid oxidation [68–70, 76]; (2) suppress inflammation via NF-κB/NLRP3 inhibition and macrophage reprogramming [73–75]; (3) normalize bile acid signaling through FXR/TGR5 and FGF19 pathways [91–93]; and (4) inhibit tumorigenic signaling via JNK and SREBP-1c suppression [69, 78, 83].

Through this multi-axis regulation, GLP-1RAs alleviate lipotoxicity, oxidative stress, and fibrosis, ultimately disrupting the metabolic and inflammatory milieu that fosters hepatocarcinogenesis. The translational coherence between in vitro mechanistic insights and in vivo outcomes strongly supports the role of GLP-1RAs as disease-modifying and potentially chemopreventive agents against HCC.

## Conclusion and future directions

The growing intersection between type 2 diabetes, MASLD, and hepatocellular carcinoma underscores a paradigm shift in how metabolic disorders are linked to liver cancer. GLP-1 receptor agonists, initially conceived as glucose-lowering drugs, now represent a promising class of agents capable of modulating several upstream drivers of hepatocarcinogenesis—including insulin resistance, steatosis, lipotoxicity, and chronic inflammation.

Across clinical trials and real-world studies, GLP-1RAs have consistently shown favorable hepatic effects and a potential to attenuate progression toward cirrhosis and HCC. Yet, available evidence remains largely observational, with signal strength varying by disease stage, comparator, and exposure duration. The absence of prospective, liver-specific randomized studies remains the main barrier to establishing causality.

Future research should, therefore, move beyond glucose-centric paradigms to focus on maintenance of metabolic liver health—integrating GLP-1RAs into broader strategies that combine pharmacologic, lifestyle, and possibly interventional approaches. Synergy with SGLT2 inhibitors, liver-directed therapies, and next-generation incretin co-agonists could further amplify hepatic and metabolic protection. Moreover, the identification of early biomarkers and imaging signatures predictive of treatment response will be essential to tailor therapy and optimize cost-effectiveness.

Ultimately, GLP-1RAs may not only transform the management of diabetes and MASLD but also redefine the landscape of primary liver cancer prevention. Their promise lies not in targeting the tumor itself, but in intercepting the metabolic and inflammatory trajectories that make the liver a fertile ground for carcinogenesis.

## Data Availability

This is not applicable to this article as no datasets were generated or analyzed in the current study.
